# HIV/AIDS knowledge in detention in Hunan province, China

**DOI:** 10.1186/1471-2458-10-221

**Published:** 2010-04-28

**Authors:** Weidong Zhang, Xinya Wang, Xi Chen, Fan Lv

**Affiliations:** 1 Office for Disease Control and Emergency Response, Chinese Center for Disease Control and Prevention, Beijing, PR China; 2 National Center for AIDS/STD Control and Prevention, Chinese Center for Disease Control and Prevention, Beijing, PR China; 3 Hunan provincial Center for Disease Control and Prevention, Changsha, PR China

## Abstract

**Background:**

Injection drug use (IDU) is one of the major modes of HIV transmission in China. Drug use is illegal in China, all identified drug users are registered by Public Security Bureau, and most were sent to detention; most detainees engaged in high risk behaviours. In order to well understand the HIV/AIDS knowledge among detainees, a survey was conducted in different detention settings in Hunan province in 2008 to assess knowledge and attitudes about HIV among detainees and to provide useful information for HIV prevention and intervention strategies in detention centers.

**Methods:**

A cross-sectional survey was conducted in 10 detentions in Hunan province, China, and demographic information along with knowledge and attitude of HIV/AIDS was collected through standardized interviews. Descriptive statistics were used to describe HIV knowledge, attitudes, and education services among detainees.

**Results:**

There were 956 detainees interviewed from 10 detention centers. The male to female ratio was 2.24:1. The majority detainees received nine years of compulsory education, accounting for 51.5%. There were nine questions to assess HIV/AIDS knowledge of detainees, and 35.7% of those surveyed answered all nine questions correctly. There were 92.3% (882/956) who consented to be informed about the HIV antibody test results when tested, and 81% (774/956) elected that their family members were also informed. All detention centers had an organized HIV/AIDS education program.

**Conclusion:**

This study gives us an overview about HIV/AIDS knowledge in detention in Hunan province, and all detention sites in the study provided HIV/AIDS intervention services among detainees that focused on HIV/AIDS knowledge, attitude, and health behaviors.

## Background

Injection drug use (IDU) is one of the major modes of HIV transmission in China [1]. Drug use is illegal in China, and all identified drug users are registered by China's Public Security Bureau. By the end of 2004, there were cumulative 1.14 million registered drug users in China [2]. Through 2004, injection drug users (IDUs) accounted for about 70% of total cumulative reported HIV infections in China [3]. By the end of 2007, there were approximately 700,000 people currently living with HIV/AIDS in China, including approximately 266,700 drug users. This accounts for 38.1% of the total number of estimated HIV cases. It is estimated that there were 50,000 new HIV infections in 2007. Of the new infections, heterosexual transmission accounts for an estimated 44.7 percent of cases and injection drug use is estimated to account for 42 percent of transmission. Sexual transmission is now the primary risk factor for HIV transmission[4].

The Chinese government has issued a series of laws and regulations to response to the HIV/AIDS epidemics and injection drug use. Prevention and intervention campaigns have addressed drug dependency and HIV, such as greatly expanding methadone maintenance therapy (MMT) and clean needle exchange programs. By the end of October 2007, 397 MMT clinics were open in 22 provinces, autonomous regions, and municipalities with 88,313 participants in the MMT treatment program. In 2006, a total of 729 needle exchange stations were established in 204 counties or districts in 17 provinces. By the third quarter of 2007, about 49,108 IDUs had participated in clean needle exchange programs [4]. Registered drug users were also sent to detention, such as detoxification and labour re-education centers in order to participate in a compulsory detoxification program [5-8].

While in detention centers, most detainees engaged in high risk behaviours, and some special characteristics of detention centers contribute to HIV transmission, such as crowded space, almost all the same gender, fighting, and other factors [9-16]. Therefore, the HIV/AIDS risk in detention is high. For the public security staff, there is also occupational HIV exposure. The same situation also existed in Chinese detention centers, especially for obligatory detoxification centers, labor detoxification centers, and female re-education centers. According to some studies, the HIV positive seroprevalence rate was between 5-10% in sentinel detention centers [17-19].

In order to better understand the trends of HIV epidemic and knowledge, attitudes and practices (KAP) about HIV among high-risk population groups, China created an HIV sentinel surveillance system in 1995, focusing efforts on groups including drug users, female sex workers, sexual transmitted diseases (STD) clinic clients, Men who have sex with men (MSM), male long distance truck drivers and students [20]. Sentinel sites for drug users and female sex workers also include obligatory detoxification centers, labor detoxification centers, and female re-education centers.

The aim of our study is to describe the results of survey among detainees in Hunan province in China conducted in 2008, to assess HIV knowledge and practices among detainees, and to provide useful information for HIV prevention and intervention strategies in detention centers.

## Methods

### Study sample

This investigation involved the public security and justice system. Specifically, the public security system included obligatory detoxification centers and custody houses, and the justice system included labor re-education centers and prison.

Detention centers were selected using stratifying sampling method, detoxification centers and custody houses were selected in the public security system, and prison and re-education centers were selected in the justice system. A Multi-level random sampling method was used to recruit individuals in different detention centers. Ten centers were randomly selected according to the system, and then at least 110 individuals were selected in every single detention center. A self-reported questionnaire was used to collect needed information.

The survey was conducted from 13 October to 30 October, 2008. Anonymity and confidentiality were guaranteed to the detainees when they participated in the survey. Under the approval of the protocol "HIV Surveillance Protocol" by the national ethical committee of China (Institutional Review Board), informed consent from participants in behavioral surveillance was required during survey [21].

Different types of detention canters were selected in order to gain better representativeness, such as centers only for women, obligatory detoxification centers, custody houses, labor re-education centers, and prison. Variables used in this study included sample source, gender, age, marital status, resident place, ethnic groups, education background, profession, time of entry detention, time space of detention, cause of detention, AIDS information, intervention and prevention services, and others.

### HIV/AIDS response in detention

All detention centers had an organized HIV/AIDS education program. Activities included seminars, workshops, and training for security staff; non-profit activities; some volunteer and some students gave courses or health education, such as "No Drug", "Health Behavior", and others. Video courses and notice boards, as well as posters, were also used for promotion of HIV/AIDS knowledge. In some detention centers, peer education and counseling were also used.

Most detention centers attempt to implement comprehensive HIV management. In one labor re-education center, Xinkaipu, a "caring center" for HIV positives started in July 2008; Baimalong established "HIV prevention unit" in July 2008. In prison, no HIV positive individuals were identified, and in a female prison, few HIV positive individuals were identified, and an established isolation caring center was implemented to provide case management collectively.

### Data analysis

The sample size included 979 samples from 10 detention centers. Epidata was used for data entry and database management. First, data cleaning was conducted, in which 23 samples were deleted. After data cleaning, a total of 956 records were included in the analysis. SPSS software (version 14.0 for Windows; SPSS Inc., Chicago, IL) was used to analyze the dataset.

## Results

### Characteristics of the study sample

There were 660 male, and 296 female, so the male to female ratio was 2.24:1. Local Hunan residents were 920, accounting for 94.4% of the sample size; transient detainees from other provinces without local permanent residence permission were 52, which accounted for 5.4%. The participants were predominantly Han Chinese, which accounted for 96.9%, 3.1% were minority ethnic groups. 47.7% samples were single, 30.6% samples married, and the remaining people were cohabiting, divorced or widowed.

The major part of detainees received nine years of compulsory education, accounting for 51.5% of the total sample size. Those with primary school or high school education were 20.0% and 21.4%, respectively. The illiteracy was 2.0%. See Table 1. Of the total, 30.2% of detainees were jobless; while job categories of the employed included farmer (24.9%), blue collar worker (12.3%), and commercial service staff (10.4%).

**Table 1:** Characteristics of sample from detentions (N = 956).

*item*		*number*	*percent (%)*
Gender	male	660	69.1
	female	296	30.9
Marital status	single	453	47.4
	married	293	30.7
	cohabited	53	5.3
	divorced	157	16.5
Age	≤ 18	43	4.5
	19-30	377	39.5
	31-40	394	41.2
	41-50	119	12.5
	>50	23	2.4
Education	Illiteracy	19	2.0
	Primary school	192	20.0
	Middle school	492	51.5
	High school	205	21.4
	College or above	48	5.0
Reason for detention	Drug use	449	47.0
	Commercial sex	16	1.5
	Others	491	51.4

Illegal drug use was the cause of detention for 47% (449/956) of the study participants; commercial sex or other reasons accounted for 51.5% (492/956).

### HIV/AIDS knowledge

There were nine questions to assess the HIV/AIDS knowledge of detainees, 35.7% of the participants answered all 9 questions correctly. The lowest correct answer rate of the nine questions was "does mosquito bite can transmit HIV virus?" (63.1% answered correctly). See Table 2. There were 1.2% of participants who had never heard HIV/AIDS.

**Table 2:** HIV/AIDS knowledge of detainees (N = 956).

*Subject*	*Correct answers*	*Correct rate (%)*
Ever heard of HIV/AIDS	945	98.8
Can a person carry HIV while still appearing healthy	781	81.7
Can HIV infected blood or blood product transmit HIV virus	900	94.1
Can sharing needles with HIV positive person transmit HIV virus	908	95.0
Can using condom correctly reduce the risk of transmission of HIV virus	682	71.3
Can an HIV infected pregnant woman transmit HIV virus to her baby	867	90.7
Can eating with an HIV infected person transmit HIV virus	779	81.5
Can a mosquito bite transmit HIV virus	603	63.1
Are people lawfully responsible if he/she transmits HIV virus intentionally	765	80.0

**Total answering all nine questions correctly**	**341**	**35.7**

### HIV/AIDS intervention services

There were 67.4% (644/956) of study participants who received HIV/AIDS information materials. The proportion of participants who received condom distribution and Voluntary Counseling and Testing (VCT) was 31.2% (298/956) and 49% (468/956), respectively.

### HIV test

There were 204 participants who had been tested for the HIV antibody before being sent to detention, which accounted for 21.3% (204/952). Of these 79.4% (162/204) knew the test results, and 36 samples were aware of HIV positive results. Most of the HIV antibody tests were conducted in a Center for Disease Control (CDC) or in other detention centers.

The total number of participants tested for HIV antibodies inside detention was 580, which accounted for 60.7% (580/951). Of these, 41.4% (240/580) participants were drug users; and 29.8% (173/580) samples knew the results. Of the 47 HIV positive participants, 36 were drug users. There were no HIV positive individuals identified among commercial sex workers.

### Test Result Notification

There were 92.3% (882/956) who wished to be notified about the test results if tested, and 81% (774/956) indicated that they wished that their family members were also notified. Of those participating in the survey, 23.4% (224/956) indicated that they would like to live with HIV positive individuals, while 55.1% (527/956) indicated that they did not like living with HIV positive individuals. There were 89% (851/956) of participants who wanted to be informed directly after the test. See Table 3.

**Table 3:** Samples' attitude of being informed of HIV test results.

	Wish to be informed of test results	Wish to have test results delivered to family member	Living with HIV positives			Time to be informed		Total
Attitude	Yes	Yes	Like	Not like	Does not matter	Directly after test	Later in other time	
Number	882	774	224	528	204	851	105	956
Percent (%)	92.3	81.0	23.4	55.1	21.3	89.0	11.0	100

### Risk behaviors

Of those interviewed in re-education centers and detoxification centers, all participants have drug use history; however, in custody houses and prisons, only a few people have a drug use history. Most drug users once shared needles, but most reported that this typically occurred at the beginning of drug use. After receiving HIV/AIDS information, needle sharing behavior almost stopped completely. Needles were typically bought in drug stores by individuals, not in clean needle exchange centers, because most clean needle exchange centers are far, and it is possible to be arrested by police. Individuals in higher social economic status (SES) levels reported using drugs at home, and did not report Needle sharing practices.

Most male detainees reported commercial sex use, and also reported sexual activity with heterosexual drug partners. Most detainees used condoms with commercial sex workers, but did not use condoms with established sex partners.

## Discussion

This study gives us an overview about HIV/AIDS knowledge in detention in Hunan province, China, but information about the HIV/AIDS risk behavior among detainees was still limited. Random sampling methods is very difficult or nearly impossible. Because of the characteristics of this population group and the operational difficulties of such investigation, legal and ethical issues should be considered [9,12-15]. Psychological factors are often neglected in China, and mental health factors should also be considered. Our finding demonstrates that the need and urgency of HIV/AIDS education campaigns in detention centers; and, our data provides an important reference for researchers and public health policy makers.

All detention sites in our study provided HIV/AIDS intervention services among detainees, and it improved HIV/AIDS knowledge, attitudes, and health behaviors among detainees. However, intervention should take action as early as possible in order to be effective and prevent further transmission [22,23]. Providing HIV/AIDS prevention and intervention services in detention centers could reduce the HIV/AIDS transmission among general population, because most detainees return to their communities after leaving detention centers. Many are at high risk for HIV/AIDS, such as drug use, commercial sex, and other factors. For HIV test result notification in detention centers, considerations should be discussed about the problematic and legal issues involved. However, test results should be provided as soon as possible, since most HIV positive individuals indicated that they would cooperate with intervention and treatment. Evidence has shown that there is a positive change in detainees' attitudes to safe sex, harm reduction, and health behaviors [23,24]. Such problems are not only public health problems, but they also need political and legal support.

Since the emergence of the HIV epidemic, China has launched an HIV prevention and harm reduction program. A comprehensive program to prevent the further transmission of HIV among drug users has been implemented that includes community based harm reduction programs, primarily a methadone maintenance treatment and needle-syringe program. In 2003, China announced a national AIDS control policy, "Four Frees and One Care" (free treatment, free Voluntary Counseling and Testing (VCT), free Prevention of Mother to Child Transmission (PMCT) and free education for AIDS orphans, and provision of social relief and care for HIV patients) [25].

## Conclusion

This study gives us an overview about HIV/AIDS knowledge in detention in Hunan province, and all detention sites in the study provided HIV/AIDS intervention services among detainees that focused on HIV/AIDS knowledge, attitude, and health behaviors. These efforts are likely to require an infrastructure that not only provides operational and financial support, but also creates an environment in which at-risk populations feel comfortable and safe in seeking help and non-discrimination.

## Competing interests

The authors declare that they have no competing interests.

## Authors' contributions

XW, WZ, XC and FL designed and conducted the analysis, WZ conducted the analysis and wrote the final version of the text. XC, FL and XW developed the questionnaire; XW collected the raw data on sites. All authors read and approved the final manuscript.

## Pre-publication history

The pre-publication history for this paper can be accessed here:

http://www.biomedcentral.com/1471-2458/10/221/prepub
